# Wellbeing Trajectories and Dementia Risk Among Mexican-Origin Adults Living in the U.S.

**DOI:** 10.1093/geroni/igaf122.1672

**Published:** 2025-12-31

**Authors:** Emily Willroth, Olivia Atherton, Angelina R Sutin, Richard Robins

**Affiliations:** Washington University in St. Louis, St. Louis, Missouri, United States; University of California Riverside, Riverside, California, United States; Florida State University College of Medicine, Tallahassee, Florida, United States; University of California, Davis, Davis, California, United States

## Abstract

Higher wellbeing is consistently associated with better cognitive health. However, little is known about how long-term trajectories of wellbeing are associated with cognitive health or modifiable dementia risk factors, particularly among populations at high risk for dementia. To address these open questions, we used data from The California Families Project, a 14-year longitudinal study of Mexican-origin adults living in the United States (analytic N = 1,082), to estimate long-term trajectories of wellbeing (i.e., life satisfaction and optimism) and whether the level and/or slope of wellbeing was associated with later cognitive health (i.e., cognitive function and impairment status, self- and informant-rated memory) and modifiable dementia risk factors (i.e., hearing loss, hypertension, higher body weight, smoking, depression, social isolation, physical inactivity, diabetes, alcohol use, poor sleep quality, inadequate or excessive sleep, inadequate health insurance, poor overall health). Higher life satisfaction level was associated with better self- and informant-reported memory, and more positive life satisfaction slope was associated with better self-rated memory. Higher optimism level was associated with better cognitive function and better self-rated memory, and more positive optimism slope was associated with better cognitive function. Life satisfaction and optimism levels and change were also associated with several modifiable dementia risk factors. These findings provide evidence that long-term trajectories of life satisfaction and optimism are associated with later levels of several cognitive health outcomes and modifiable dementia risk factors in Mexican-origin adults living in the United States, an ethnic group that experiences disproportionate rates of dementia.

